# Integrated analyses for identification of a three‐gene signature associated with Chaihu Shugan San formula for hepatocellular carcinoma treatment

**DOI:** 10.1111/jcmm.18211

**Published:** 2024-04-13

**Authors:** Jia‐heng Xing, Ru‐xue Tan, Fei‐er Huang, Nan Tian

**Affiliations:** ^1^ College of Life Science Zhejiang Chinese Medical University Zhejiang Hangzhou China

**Keywords:** Chaihu Shugan San, hepatocellular carcinoma, immunotherapy, molecular docking, Surface Plasmon Resonance analysis

## Abstract

Chaihu Shugan San (CSS) is a well‐known traditional herbal formula that has the potential to ameliorate hepatocellular carcinoma (HCC); however, its mechanism of action remains unknown. Here, we identified the key targets of CSS against HCC and developed a prognostic model to predict the survival of patients with HCC. The effect of CSS plus sorafenib on HCC cell proliferation was evaluated using the MTT assay. LASSO‐Cox regression was used to establish a three‐gene signature model targeting CSS. Correlations between immune cells, immune checkpoints and risk score were determined to evaluate the immune‐related effects of CSS. The interactions between the components and targets were validated using molecular docking and Surface Plasmon Resonance (SPR) assays. CSS and sorafenib synergistically inhibited HCC cell proliferation. Ten core compounds and 224 targets were identified using a drug compound–target network. The prognostic model of the three CSS targets (AKT1, MAPK3 and CASP3) showed predictive ability. Risk scores positively correlated with cancer‐promoting immune cells and high expression of immune checkpoint proteins. Molecular docking and SPR analyses confirmed the strong binding affinities of the active components and the target genes. Western blot analysis confirmed the synergistic effect of CSS and sorafenib in inhibiting the expression of these three targets. In conclusion, CSS may regulate the activity of immune‐related factors in the tumour microenvironment, reverse immune escape, enhance immune responses through AKT1, MAPK3, and CASP3, and synergistically alleviate HCC. The co‐administration of sorafenib with CSS has a strong clinical outlook against HCC.

## 1 INTRODUCTION

Hepatocellular carcinoma (HCC) is one of the most common clinical malignancies and a leading cause of death worldwide.^1^, ^2^ It is associated with high morbidity, mortality and metastasis. Currently, the treatments for HCC have progressed from a single option of surgical resection to a spectrum of options, including locoregional ablation, transcatheter arterial chemoembolization (TACE), systemic molecular‐targeted therapies and/or immunotherapy. However, locoregional ablation and TACE can easily cause local recurrence and post‐embolism syndrome.^3^ Further, immunotherapy and molecular‐targeted medications are vulnerable to drug resistance.^4^ Currently, there is no consensus regarding adjuvant treatment for patients with HCC after resection. Therefore, determining effective and safe adjuvant therapies for patients is still a great challenge in clinical practice.

Traditional Chinese Medicine (TCM) has become an integral component of cancer prevention and treatment.^5^, ^6^ Rigorous clinical studies have shown that TCM decoctions inhibit HCC growth, reduce the risk of recurrence, and improve treatment resistance by mitigating toxic side effects and/or increasing dose sensitivity.^4^ Chaihu Shugan San (CSS) is a TCM decoction containing *Bupleurum chinense* DC. (Chaihu, CH), *Citrus reticulata* Blanco (Chenpi, CP), *Ligusticum striatum* DC. (Chuanxiong, CX), *Paeonia lactiflora* Pall. (Baishao, BS), *Citrus aurantium* L. (Zhiqiao, ZQ), *Cyperus rotundus* L. (Xiangfu, XF) and *Glycyrrhiza uralensis* Fisch. (Gancao, GC). It has been used clinically as an adjuvant treatment for HCC in China. It exerts beneficial effects at different stages of the disease, including reducing recurrence and metastasis after surgery, attenuating the adverse effects of conventional tumour treatments, preserving liver function and alleviating clinical symptoms.^7^, ^8^ However, because of the synergistic action of multiple components and targets, it is difficult to fully elucidate the mechanisms of CSS, limiting its clinical application.

**FIGURE 1:**
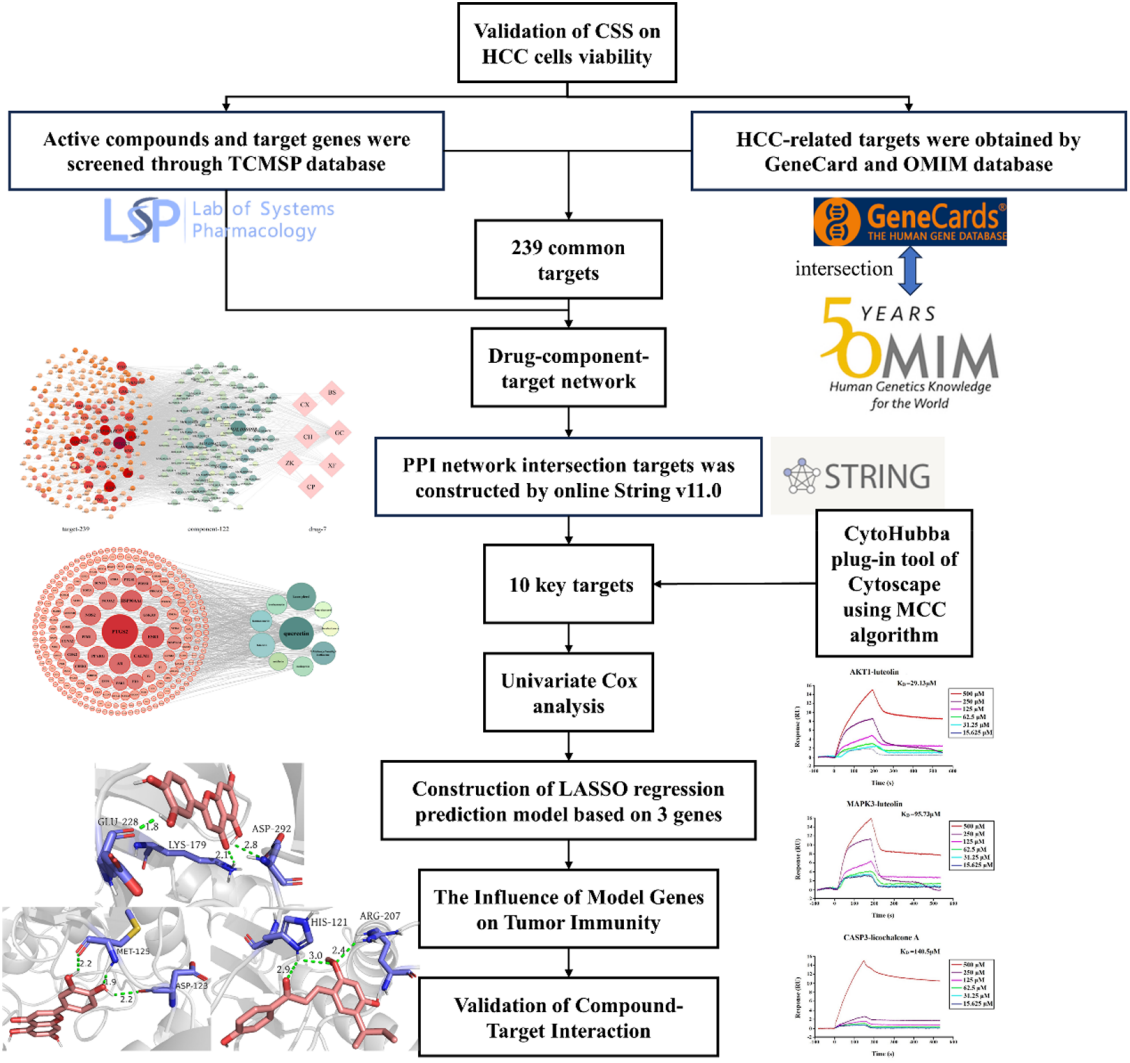
Flow chart of the approach used in the study.

Therefore, to explore the mechanism of action of CSS against HCC, we screened its active components and related targets for HCC treatment using network pharmacology. Next, we screened three targets related to the prognosis of patients with HCC as hub genes to construct a CSS three‐gene target model and evaluated its predictive potential using LASSO‐Cox regression analysis. The correlation between model‐based risk scores and tumour‐infiltrating immune cells was determined to explore the immune‐related effects of CSS. Molecular docking and Surface Plasmon Resonance (SPR) analyses were performed to verify binding between the three target genes and the corresponding active components of CSS. Our study provides a systematic view of the potential mechanism of CSS in patients with HCC (Figure 1).

## 2 MATERIALS AND METHODS

### 2.1 Preparation of CSS water extraction

All Chinese herbal medicines were purchased from Chinese medicine outpatient clinics of Zhejiang Chinese Medical University. The raw herbal material was grinded into powder and extracted twice with boiling distilled water (1:10, w/v) under reflux, 2 h each time. The CSS water extract was then filtered and evaporated to a concentration of 1 g of crude drug per mL and stored at 4°C.

### 2.2 Cell culture

Hep3B and HepG2 cells (ATCC, Manassas, VA) were cultured using Dulbecco's Modified Eagle's Medium (DMEM, Cienry, Zhejiang, China) with 10% heat‐inactivated fetal bovine serum (sijiqing, Zhejiang, China) and 1% penicillin–streptomycin (biosharp, Beijing, China) solution at 37°C in a humidified incubator containing 5% CO_2_.

### 2.3 Cell viability

HCC cells were seeded in 96‐well plates at an initial density of 3 × 10^3^ cells/well, and then treat with different doses of CSS (0.05, 0.5, 5, 50, 500 μg/mL) with or without sorafenib (5 μM). The cultures were incubated at 37°C in a humidified incubator containing 5% CO_2_. Following MTT (Solarbio, Beijing, China) solution (5 mg/mL, 10 μL/well) was added for 4 h. After discarding the supernatant, 150 μL DMSO (Sigma‐Aldrich, Shanghai, China) was added to each well, and the OD_490_ was detected using the microplate reader (RT‐6000 analyser, Rayto, Shenzhen, China).

### 2.4 Screening active compounds and target genes

The composition of the CSS was selected using the Traditional Chinese Medicine Systems Pharmacology Database (TCMSP, https://old.tcmsp‐e.com/tcmsp.php). The oral bioavailability (OB) was set to ≥30% and drug‐likeness (DL) was set to ≥0.18. The targets of the components in CSS were screened out from the Uniprot (https://www.uniprot.org/) and NCBI (https://www.ncbi.nlm.nih.gov/) database, and the HCC‐related targets were screened out from the GeneCards (https://www.genecards.org/) and OMIM (https://omim.org/) database.

### 2.5 Compound‐target network construction

The compound‐target network of CSS was constructed using Cytoscape 3.8.0. The active compounds and intersection targets were used to create network nodes, and compound‐target connections were represented by the edges of the network nodes.

### 2.6 Construction of protein–protein interaction (PPI) network

The protein interaction relationship was obtained through the search tool for the Retrieval of Interacting Genes/Proteins (STRING, https://string‐db.org/) online database, the minimum required interaction score was set to ≥0.4. The PPI network of targets was constructed using Cytoscape 3.8.0. The key nodes in the interaction network were obtained by using the Cytoscape plug‐in tool CytoHubba.

### 2.7 LASSO‐Cox regression prediction model

The prognostic value of the hub genes was determined by univariate Cox regression analysis, and *p* < 0.05 was considered statistically significant. Cox regression analysis was performed using the “glmnet” R package to further identify genes related to HCC prognosis. Based on the median risk score from LASSO's regression prognosis model, patients are divided into low‐ and high‐risk groups. The Kaplan–Meier analysis of overall survival (OS) was executed using the ‘survfit’ R package, and logrank tests were used to determine the significance between survival curves. Receiver operating characteristic (ROC) curves were generated using the R package “pROC” (version 1.17.0.1) to evaluate the accuracy of the risk score model.

### 2.8 Correlation between the prognostic model and tumour immunity

The ‘CIBERSORT’ R package was used to download immune cell content data. To explore the correlation between the risk scores and tumour immunity, we mapped the correlation plots between the risk scores and immune cells, and between the risk scores and immune checkpoints.

### 2.9 Molecular docking analysis

Molecular modelling was performed using AutoDock Vina,^9^, ^10^ 3D and 2D representations of docking complexes are rendered with the help of PyMOL (DeLano Scientific LLC, San Francisco, CA) and Discovery Studio (BIOVIA, San Diego, CA) programs, respectively. The AlphaFold Protein Structure Database (https://alphafold.ebi.ac.uk/) was used to obtain key targets used for docking. Pubchem (https://pubchem.ncbi.nlm.nih.gov/) was used to obtain the structures of drugs. The interaction between the protein and drugs was studied with the help of scores.

### 2.10 Surface Plasmon Resonance (SPR) analysis

Plexera Nanocapture™ Chip (Plexera LLC, Seattle, WA) was used in this work. Phosphate‐buffered saline was used as the running buffer and 10 mM NaOH as the regeneration buffer. Multiple concentration groups were set for each candidate compound in the mobile phase sample. PlexArray HT system (Plexera, LLC, Seattle, WA) was used to record binding signals. The dissociation constant, dissociation rate constant and association rate constant were carried out by Plexera Data Explorer.

### 2.11 Western Blot (WB) analysis

Proteins were electrophoresed by SDS‐PAGE and transferred to PVDF membrane (Merck Millipore, MA, USA). After blocked with a 1% BSA solution, the membranes were incubated overnight at 4°C with the following primary antibodies: anti‐AKT1 (1:5000, ET1609‐47; HUABIO, Hangzhou, China), anti‐CASP3 (1:2000, ET1602‐39; HUABIO, Hangzhou, China), anti‐MAPK3 (1:1000, R24246; Zenbio, Chengdu, China), or anti‐gapdh (1:5000, ABL1020; Abbkine, Wuhan, China). Then, the membranes were incubated with goat anti‐mouse or anti‐rabbit antibody (Dawen Biotec) at room temperature for 2 h. The ECL chemiluminescence method was used.

### 2.12 Statistical analysis

All data were statistically analysed using GraphPad Prism 8.0 (GraphPad Software, Inc., La Jolla, CA). The experimental data were presented as the mean ± standard deviation (SD). Statistical analysis was performed for normal distribution using the Shapiro–Wilk test. Comparisons between two groups were performed using Student's *t*‐test, and multiple comparisons were analysed using one‐way analysis of variance. Correlation analysis was performed using Pearson's correlation analysis. *p* < 0.05 was considered statistically significant.

## 3 RESULTS

### 3.1 CSS plus sorafenib inhibited HCC cell viability

**FIGURE 2:**
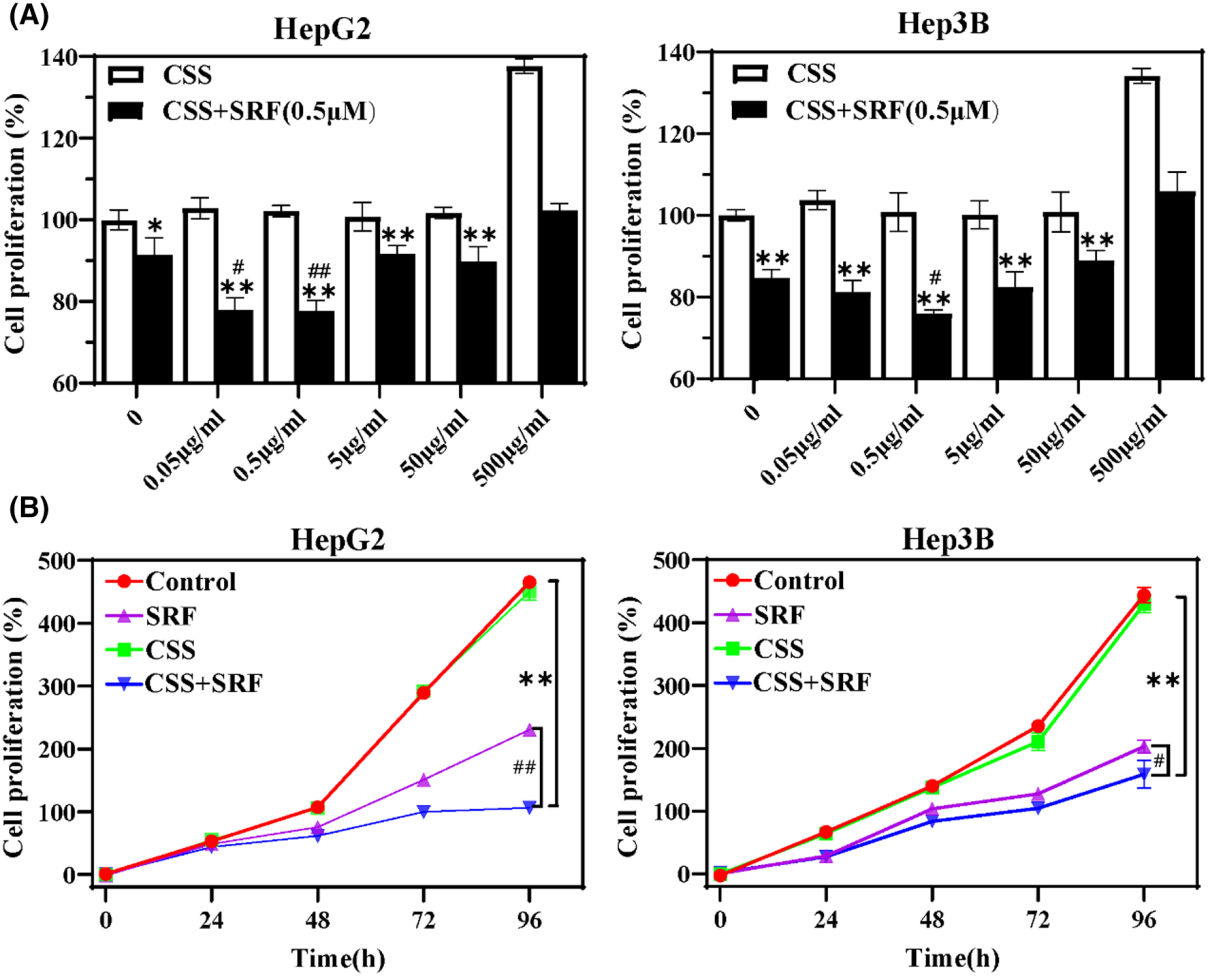
Synergistic effect of CSS on sorafenib‐inhibited HCC cell proliferation. (A) Cell viability of Hep3B/HepG2 cells exposed to sorafenib combined with different doses of CSS (0.05, 0.5, 5, 50 and 500 μg/mL) for 24 h. (B) Cell viability of Hep3B/HepG2 cells treated with CSS combined with sorafenib at different times (24, 48, 72 and 96 h). HCC cells cultured without CSS or sorafenib were defined as the control group. **p < 0.01 versus control group; #*p* < 0.05 and ##*p* < 0.01 versus sorafenib group.

The MTT assay was performed 48 h after treatment with different doses of CSS with or without sorafenib. CSS alone had no obvious cytotoxicity on both Hep3B and HepG2 cells at concentrations of 0.05–500 μg/mL, while when combined with sorafenib, it significantly reduced the cell viability of HCC cells, with 0.5 μg/mL CSS plus sorafenib causing the most significant decrease in Hep3B and HepG2 cell viability (Figure 2A). Owing to this, 0.5 μg/mL CSS was used in subsequent experiments. As shown in Figure 2B, the decrease in cell viability was greater with longer drug exposure. The sorafenib + CSS group had the most significant cell viability inhibition rates in Hep3B and HepG2 cells at 72 and 96 h, which were 84.47 ± 0.99% and 77.02 ± 3.44%, respectively. These findings indicate that CSS has a synergistic effect on sorafenib‐inhibited HCC cell proliferation.

### 3.2 Active compounds in CSS and their potential targets

**FIGURE 3:**
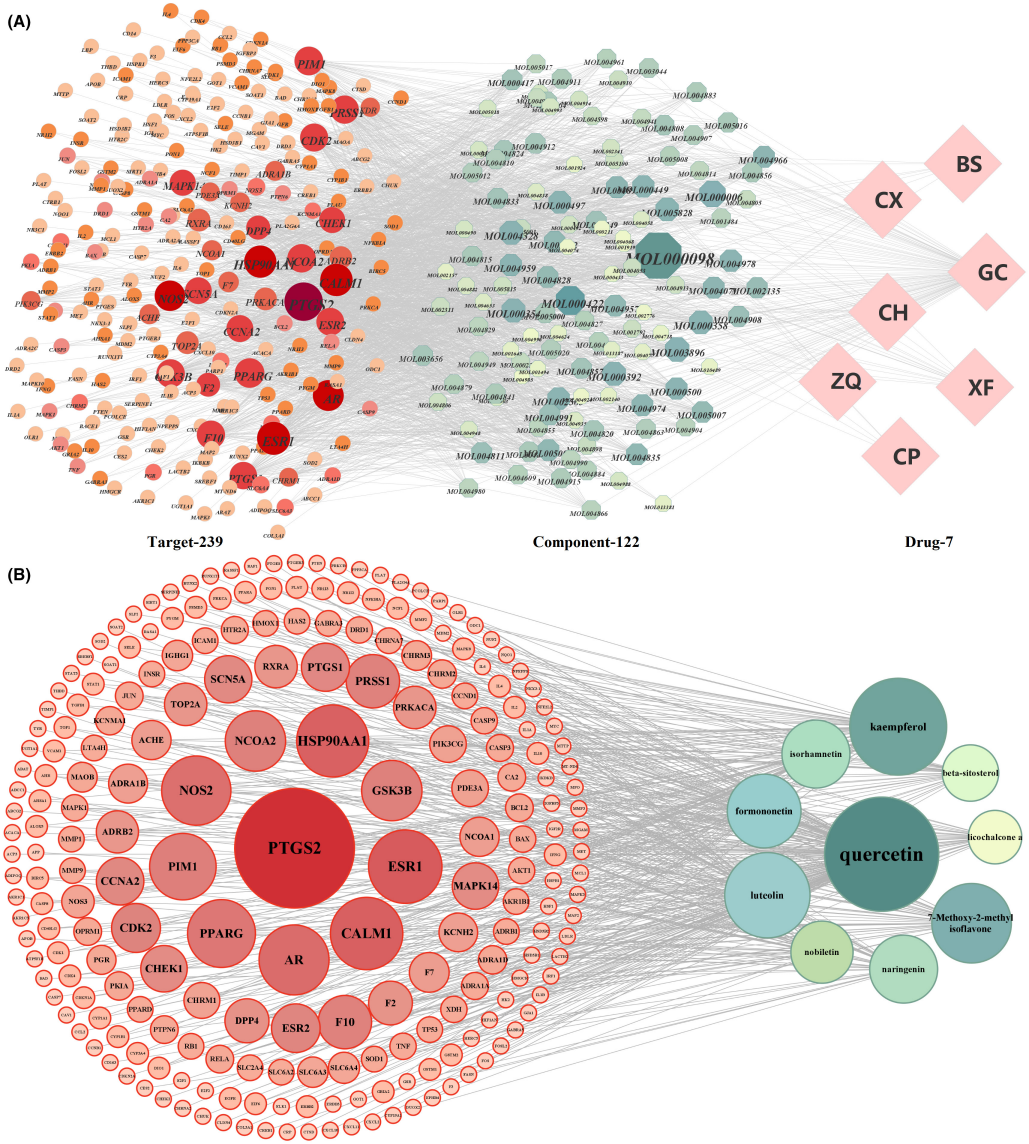
Network pharmacology prediction of HCC treatment with CSS. (A) ‘Drug‐component‐target’ network of CSS in the treatment of HCC. (B) ‘Compound‐target’ networks of 10 core compounds and targets. The darker the colour and larger node size depicts higher degree values.

The active compounds in CSS and their target genes were screened using the TCMSP database. After removing repeated names, 122 active compounds and 239 target genes were obtained: 17 components and 184 genes from CH, 5 components and 64 genes from CP, 7 components and 30 genes from CX, 13 components and 88 genes from BS, 5 components and 90 genes from ZQ, 18 components and 212 genes from XF, and 92 components and 224 genes from GC. Next, 13,488 HCC‐related targets were obtained from GeneCards and OMIM databases. Among these, 239 common targets were shared between potential targets of CSS and HCC. Subsequently, drug component‐target networks were constructed using Cytoscape 3.8.0. (Figure 3A). The top 10 components by degree value were regarded as the main active components of CSS (Table 1). These 10 core compounds and their 224 targets were also used to produce a plot of compound‐target networks, including 234 nodes and 489 edges (Figure 3B).

**TABLE 1:** The main effective components of CSS in the treatment of HCC.

MOL ID	Active components	OB (%)	DL	Molecular structure	Degree value	Compound source
MOL000098	Quercetin	46.43334812	0.27525	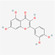	139	CH, XF, GC
MOL000422	Kaempferol	41.88224954	0.24066	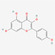	61	CH, XF, BS, GC
MOL000006	Luteolin	36.16262934	0.24552	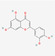	51	XF
MOL003896	7‐Methoxy‐2‐methyl isoflavone	42.56474148	0.19946	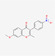	42	GC
MOL000392	Formononetin	69.67388061	0.21202	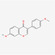	39	GC
MOL004328	Naringenin	59.29389773	0.21128	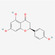	40	CP, ZQ, GC
MOL000354	Isorhamnetin	49.60437705	0.306	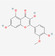	38	CH, XF, GC
MOL005828	Nobiletin	61.66943932	0.51652	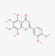	36	CP, ZQ
MOL000358	Beta‐sitosterol	36.91390583	0.75123	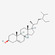	37	XF, ZQ, BS
MOL000497	Licochalcone A	40.78965199	0.28517	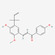	33	GC

Abbreviations: BS, baishao; CH, chaihu; CP, chenpi; DL, drug‐like Pproperties; GC, gancao; OB, oral bioavailability; XF, xiangfu; ZQ, zhiqiao.

### 3.3 GO and KEGG enrichment analyses

**FIGURE 4:**
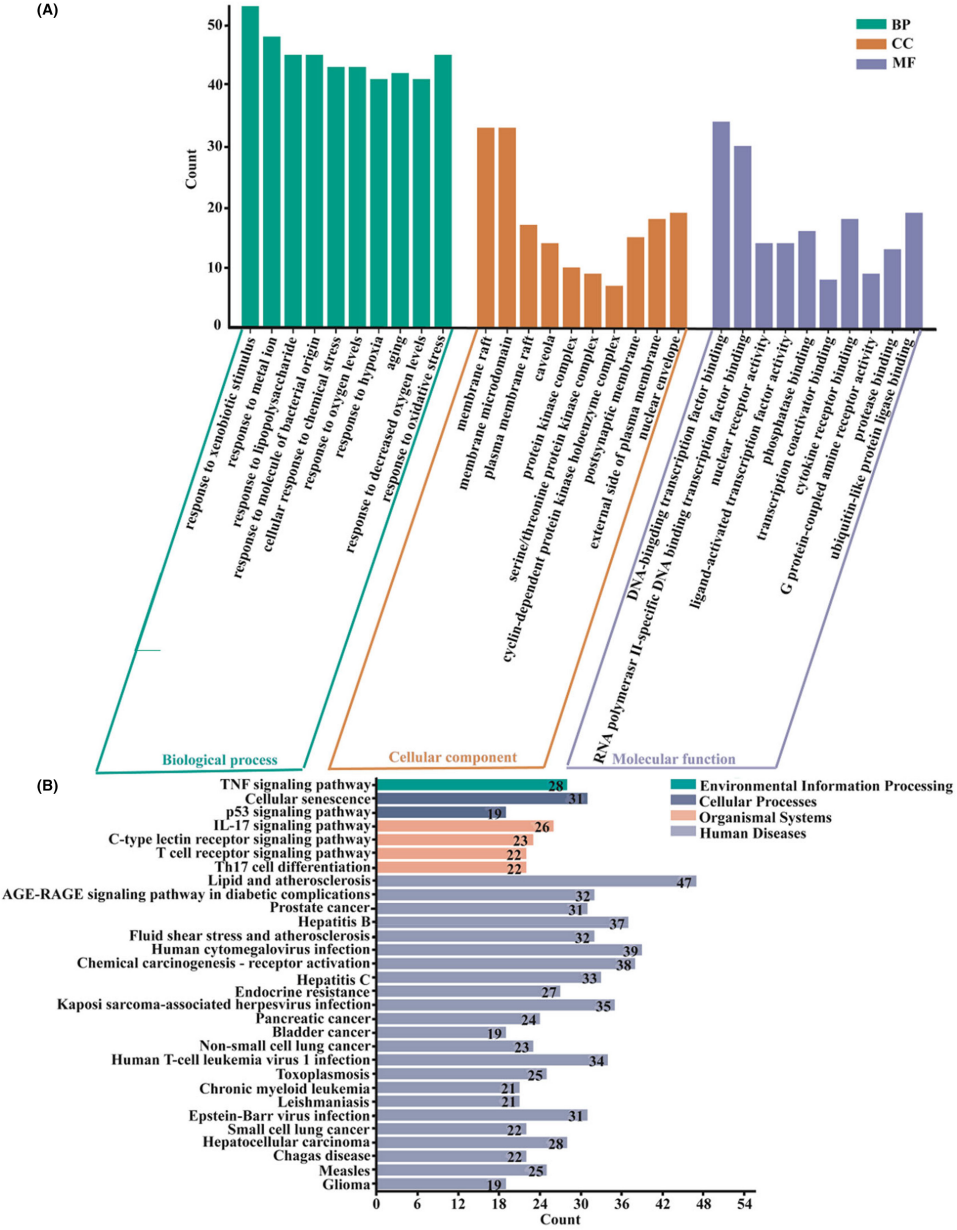
GO and KEGG enrichment analysis. (A) GO functional and (B) KEGG pathway enrichment histogram.

To understand the physiological processes and biological functions of the intersecting targets in CSS macroscopically and comprehensively, GO functional enrichment and KEGG pathway enrichment analyses were performed. The GO results in Figure 4A show that these targets favoured important biological processes, such as the response to extracellular environment stimulation (xenobiotic stimulus, metal ions, and lipopolysaccharide) in BP, membrane raft and protein kinase in CC, and transcription factor binding, nuclear receptor activity, and ligand‐activated transcription factor activity in MF. KEGG pathway analysis indicated that immunological signalling pathways (including the IL‐17 signalling pathway, T cell receptor signalling pathway and Th17 cell differentiation), TNF signalling pathway, p53 signalling pathway, lipid atherosclerosis, hepatitis B and hepatitis C were enriched, as shown in Figure 4B (corrected *p* < 0.05).

### 3.4 Analysis of CSS key targets in the treatment of HCC

**FIGURE 5:**
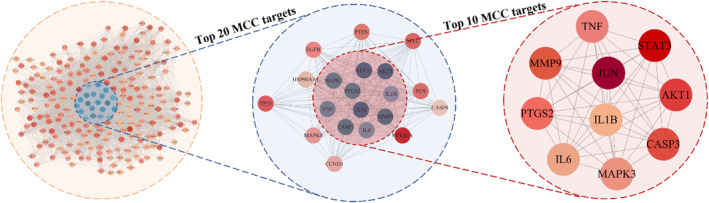
Top 10 Maximal Clique Centrality (MCC) targets of CSS for primary liver cancer.

The physiological functions of proteins are typically regulated by their interactions and corresponding pathways.^11^ To illustrate the functions and mechanisms of CSS in HCC treatment, a PPI network of 224 intersection targets was constructed using STRING v11.0. The key targets of CSS were identified using the CytoHubba plug‐in tool of Cytoscape and the Maximal Clique Centrality (MCC) algorithm. Genes with the top 20 linkage degrees were identified and an interactive subnetwork was constructed (Figure 5). Using similar screening criteria, genes with the top 10 linkage degrees were identified and defined as key targets (AKT1, CASP3, IL1B, IL6, JUN, MAPK3, MMP9, PTGS2, STAT3 and TNF) (Figure 5).

### 3.5 Construction of the LASSO regression prediction model

**FIGURE 6:**
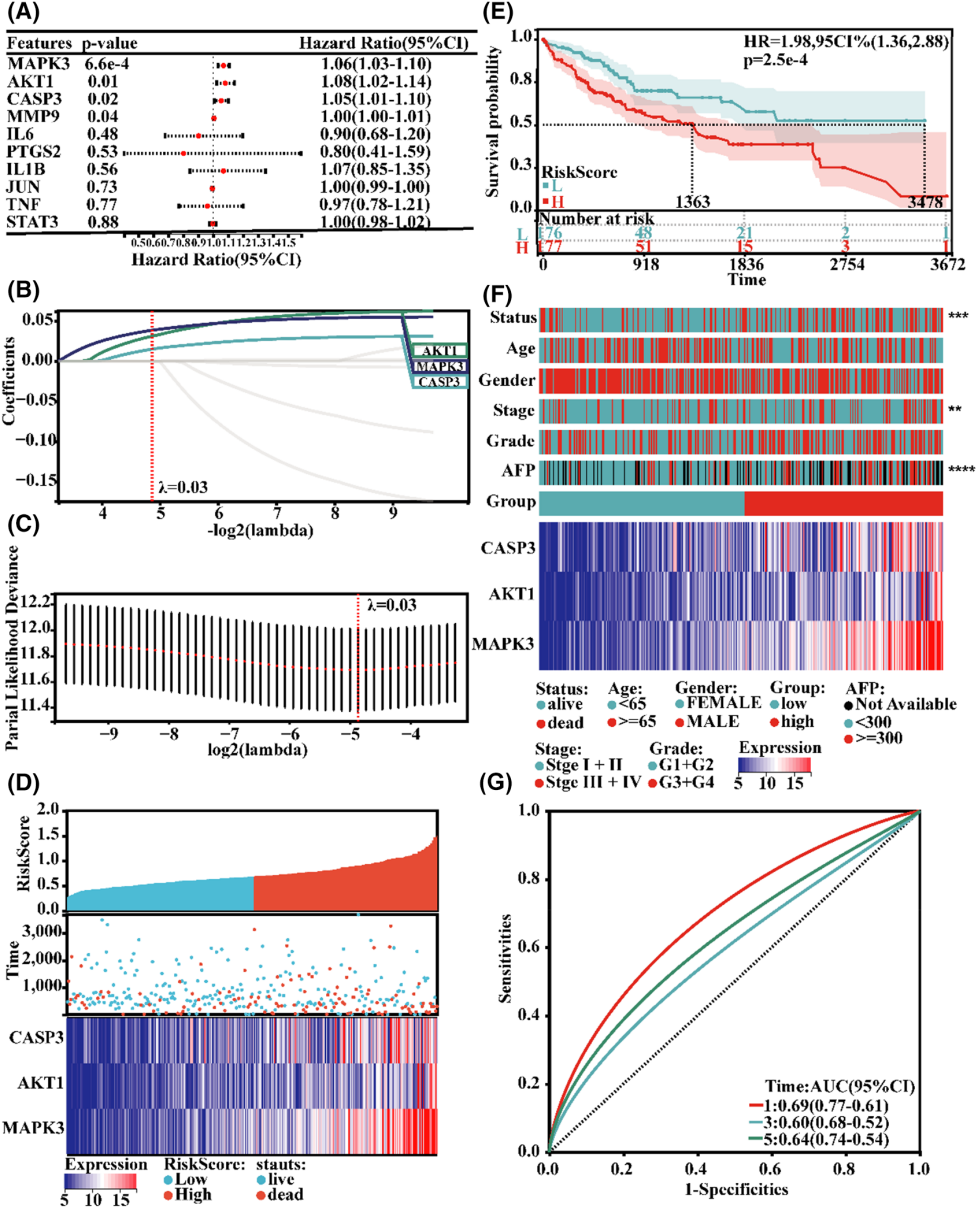
Construction and prognostic value of the prognostic model of CSS target genes. (A) Univariate Cox analysis of network core genes. (B, C) LASSO prognostic regression model. (D) The distribution of the risk scores, survival status and expression of the three critically predictive genes. (E) Survival curves of the high‐ and low‐risk groups in the TCGA database. (F) Clinical information and heatmap of the expression levels of the three model genes in the high‐ and low‐risk groups. (G) ROC curve verifying the accuracy of risk.

To further select key clinically related targets and evaluate their prognostic roles, univariate Cox regression analysis was initially performed using transcriptome data and clinical information from the TCGA‐LIHC database. Among the 10 key targets, expression of CASP3, AKT1 and MAPK3 was associated with OS in patients with HCC (Figure 6A).

We constructed a three‐gene signature for survival prediction using LASSO‐Cox regression analysis, and the risk score was calculated using the coefficients of CASP3, AKT1 and MAPK3 (Figure 6B,C). The resultant equation was as follows: Risk score = 0.014946834324991 * CASP3 + 0.0310975867942454 * AKT1 + 0.0385177274200257 * MAPK3. Based on the median risk score, patients in the TCGA‐LIHC database were divided into low‐ and high‐risk groups. The results in Figure 6D,E show that patients with higher risk scores had more deaths or shorter survival times (median OS: 1363 days vs. 3478 days, *p* < 0.001). The heat map in Figure 6F shows the relative expression of these three genes and the clinical characteristics of the two groups. High‐risk scores indicate higher histological grades, higher AFP levels and poorer outcomes. Furthermore, the ROC curves show a reliable predictive ability for the three‐gene signature, and the areas under the curve for 1, 3, and 5‐years were 0.69, 0.60 and 0.64, respectively (Figure 6G).

**FIGURE 7:**
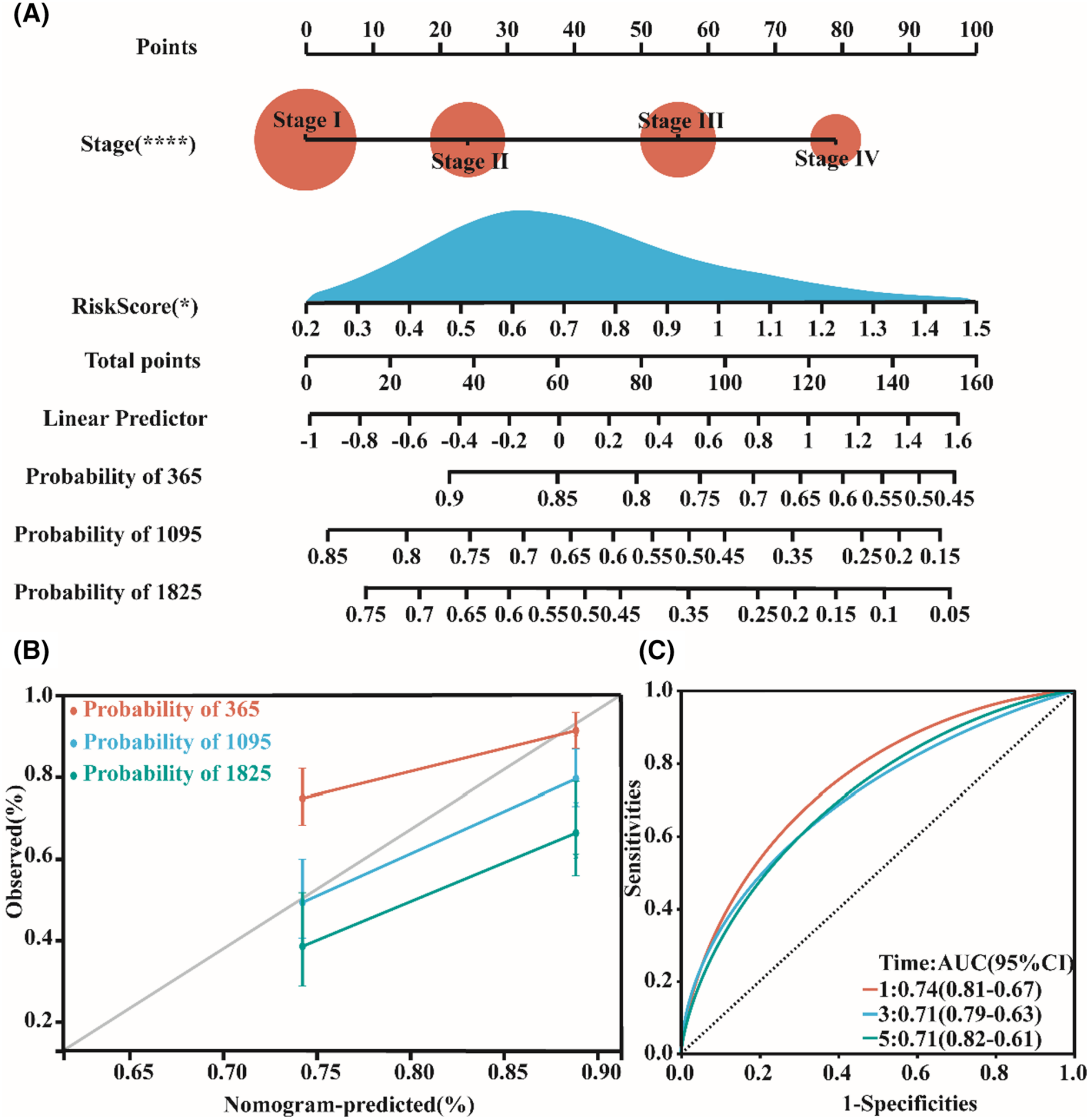
Validation of the prognostic model of CSS target genes. (A, B) Prediction of patient survival using a nomogram. (C) ROC curve verifying the accuracy of risk.

We also integrated a three‐gene‐based risk score and stage to construct a nomogram for predicting the 1‐, 3‐, and 5‐year OS of patients with HCC (Figure 7A,B). The prediction accuracy of the nomogram was 1:0.74 (0.81–0.67), 3:0.71 (0.79–0.63) and 5:0.71 (0.82–0.61), showing good survival probability prediction of the model (Figure 7C).

### 3.6 Influence of model genes on tumour immunity

**FIGURE 8:**
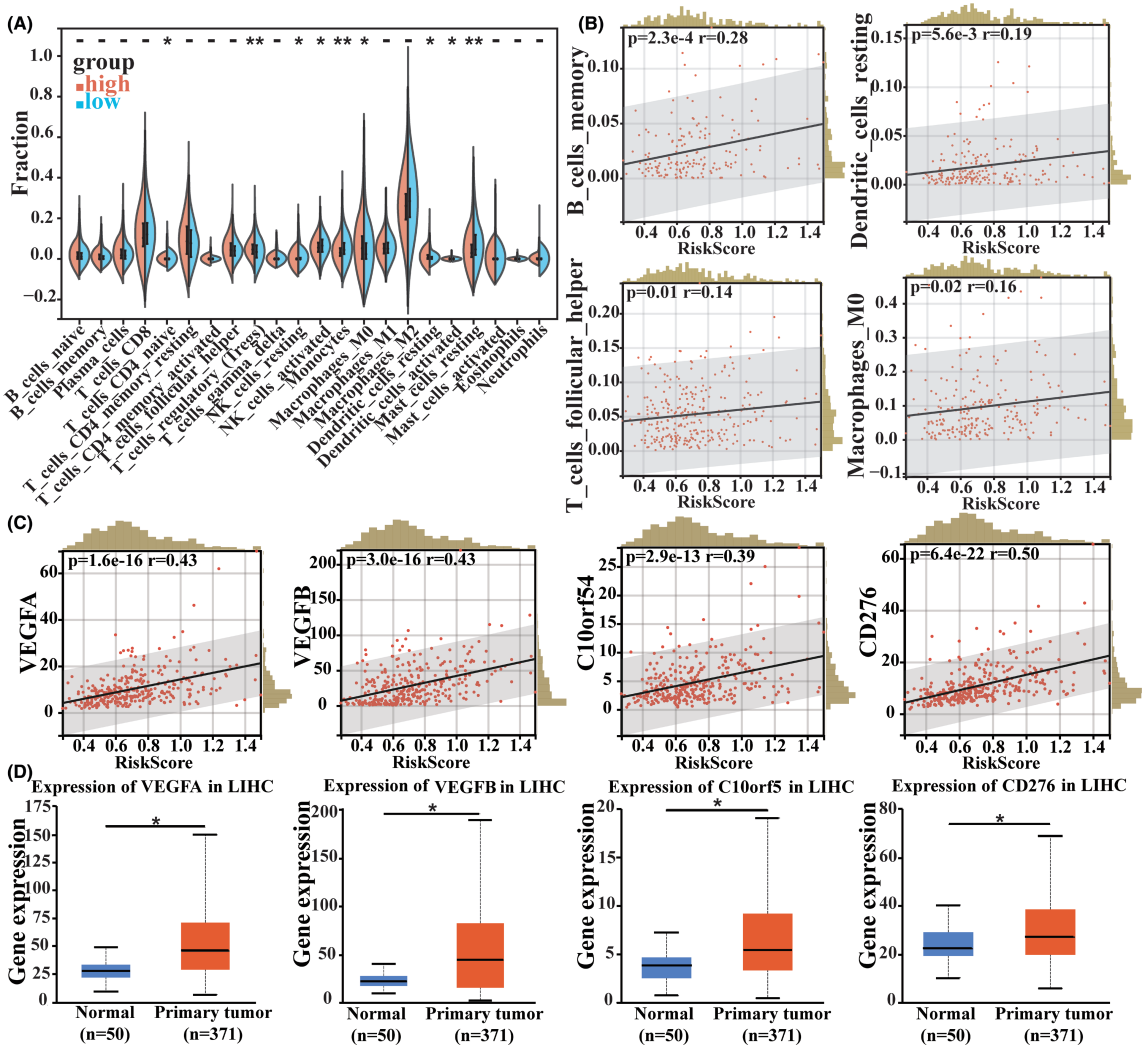
Analysis of the correlation between the risk scores and immune cells or major immunosuppressive genes. (A) Split violin plot of various immune cell contents in the TCGA‐LIHC database. (B) Linear regression of B cell memory, DC resting, follicular helper T cell and M0 macrophage levels, and risks. (C) Linear regression between expression levels of various immune checkpoints and risk scores. (D) Boxplot of the expression levels of various immune checkpoints between normal and tumour samples. *p < 0.05, **p < 0.01.

Next, we investigated whether the CSS‐targeted three‐gene signature influenced immune response in patients with HCC. CIBERSORT was utilized to analyse the 22 immune cell compositions in the low‐ and high‐risk groups. The results showed that the concentrations of naïve CD4+ T cells, activated NK cells and monocytes were significantly reduced in the high‐risk group, whereas regulatory T cells and M0 macrophages were relatively increased (Figure 8A). Subsequently, we investigated the correlation between the risk score and the content of various types of immune cells. As shown in Figure 8B, memory B cells, M0 macrophages, dendritic resting cells and follicular helper T cells were positively correlated with the risk score. We also observed correlations between risk scores and several immune checkpoints. The risk score strongly correlated with the expression of VEGFA, VEGFB, CD276 and C10orf54 (Figure 8C), and each immune checkpoint was differentially expressed between normal and tumour samples (Figure 8D). Taken together, our results suggest that heterogeneous immune infiltration in high‐ and low‐risk patients with HCC might be a prognostic indicator and target of immunotherapy and may have important clinical significance.

### 3.7 Validation of compound‐target interaction by molecular docking and SPR assay

**FIGURE 9:**
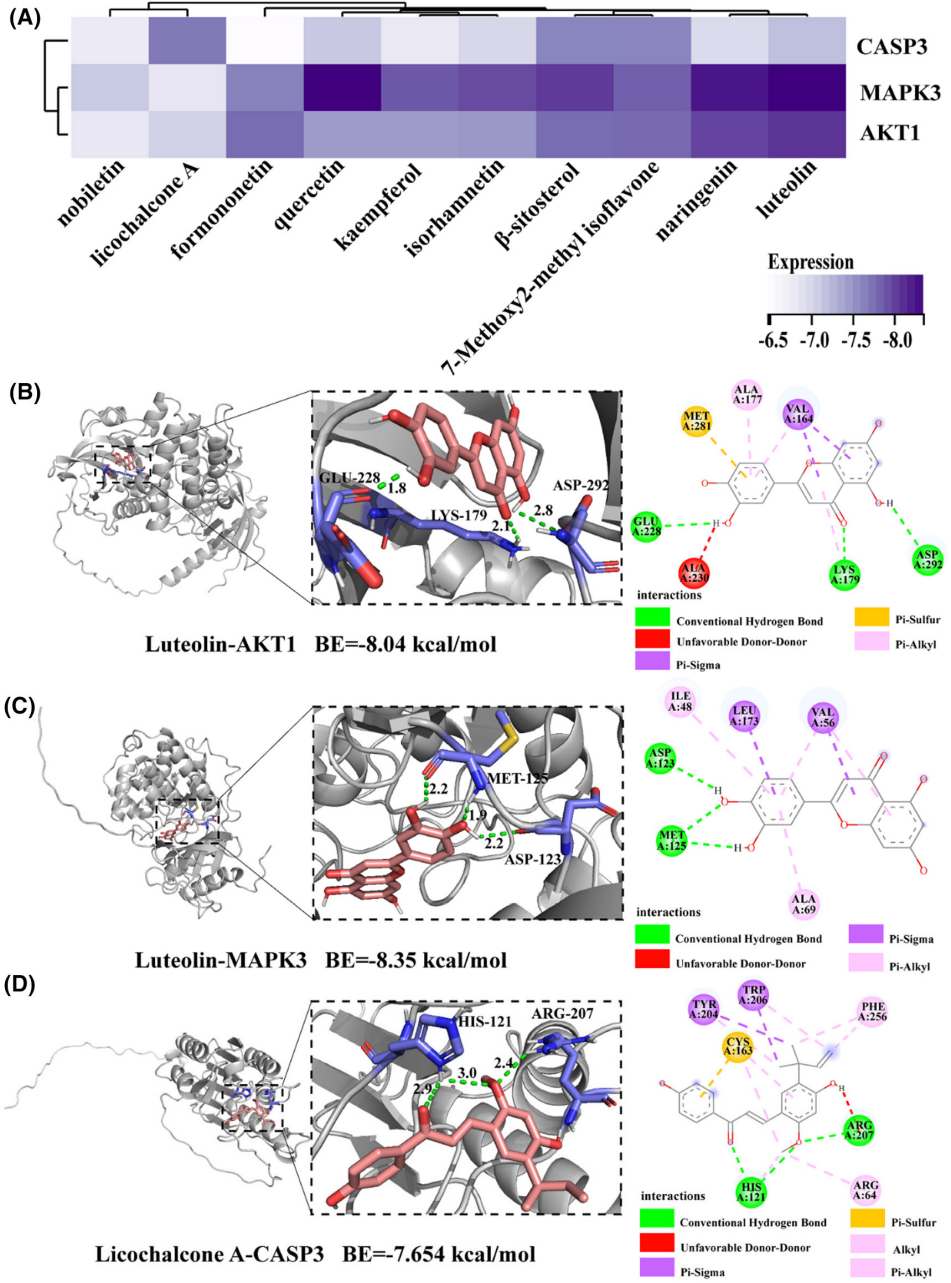
Molecular docking diagram of CSS active components and target genes. (A) Heat map of binding energy (BE) of the interaction between CSS components with key targets. The darker the colour depicts a lower BE. (B–D) 3D and 2D images of the interaction between CSS components with key targets.

**FIGURE 10:**
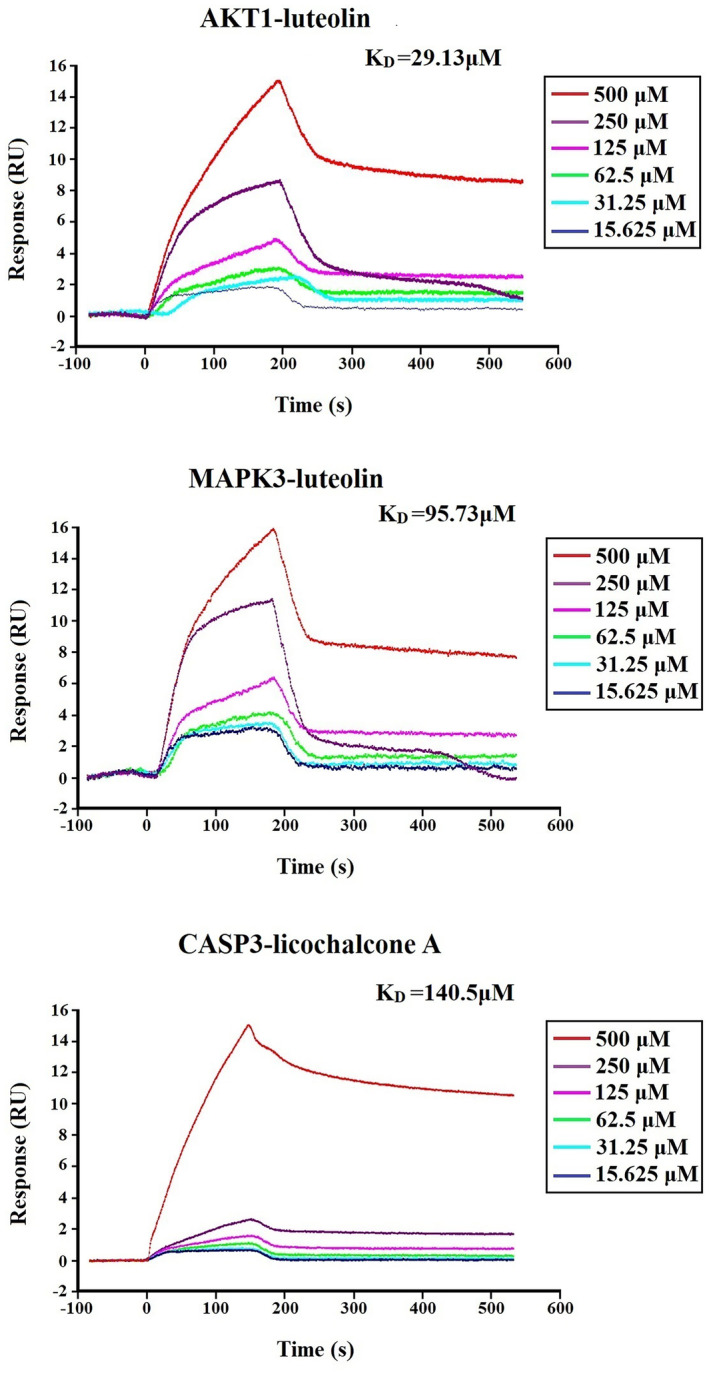
SPR analyses of the interaction between CSS components with key targets.

To explore the binding capacity between the three hub genes and the 10 core compounds of CSS, molecular docking was performed using AutoDock Vina. According to the heatmap of binding energy (BE) (Figure 9A), three genes exhibited binding capacity to most of the active compounds (score ≤−6); MAPK3 and AKT1 showed very strong binding activity to luteolin (score ≤−8). The docking modes showed that luteolin formed a hydrogen bond with MAPK3 through ASP‐123 and MET125, and with AKT1 through GLU‐228, ASP‐292, and LYS‐179; licochalcone A formed a hydrogen bond with CASP3 through HIS‐121 and ARG‐207 (Figure 9B–D). The binding activities of MAPK3‐luteolin, AKT1‐luteolin, and CASP3‐licochalcone A were determined using an SPR assay. As shown in Figure 10, AKT1 and MAPK3 had strong binding affinity with luteolin, with KD values of 29.13 μM and 95.73 μM, respectively. Licochalcone A also exhibited binding affinity for CASP3.

### 3.8 CSS plus sorafenib inhibited the expression of hub targets in HCC cells

**FIGURE 11:**
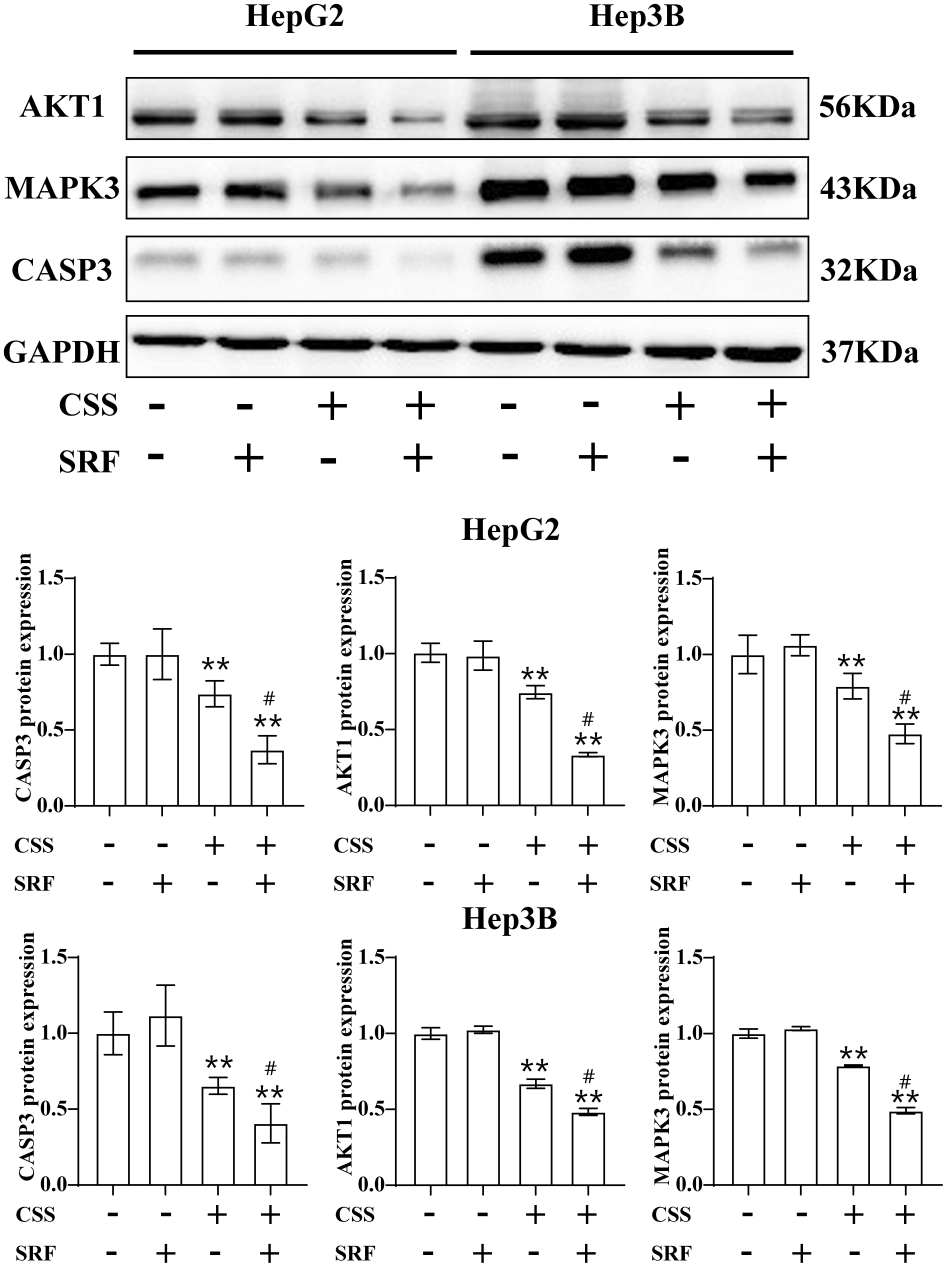
The expression levels of CASP3, AKT1 and MAPK3 in HCC cells treated with CSS or sorafenib alone, and CSS combined with sorafenib. **p < 0.01 versus control group; #*p* < 0.05 and ##*p* < 0.01 versus CSS group.

The western blot analysis was employed to detect the expression of AKT1, MAPK3 and CASP3 after treatment with CSS or sorafenib alone, and CSS combined with sorafenib. The results showed that treatment with 0.5 μM sorafenib did not change the expression levels of CASP3, AKT1 and MAPK3 significantly; 0.5 μg/mL CSS treatment reduced the protein levels of CASP3, AKT1 and MAPK3, while treatment with CSS combined with sorafenib markedly increased the reduction of CASP3, AKT1 and MAPK3 expression compared to treatment with CSS alone, confirming the effectiveness of these three targets (Figure 11).

## 4 DISCUSSION

CSS has been widely recognized for its pharmacological effects, including analgesic, liver‐protective, anti‐tumour, anti‐inflammatory, antidepressant, and anti‐fibrosis properties.^12^, ^13^, ^14^, ^15^, ^16^ In a previous study of CSS adjuvant therapy, 84 patients were randomly divided into a control group (standard treatment with sorafenib) and an experimental group (treatment with CSS and sorafenib). After 6 months of treatment, tumour reduction in the experimental group was higher and the mortality rate was remarkably lower than that in the control group.^17^ Furthermore, the inhibitory effects of CSS in combination with sorafenib on serum tumour markers (AFP and CA19‐9) and liver function markers (ALT and TBIL) were stronger than those of sorafenib alone. Yan et al. (2019) reported that CSS combined with TACE further reduced tumour volume, lowered recurrence rate, increased the 1‐year survival rate and reduced adverse reactions compared to TACE alone. However, there are no reports on the effects of CSS on HCC cells. In this study, we found, for the first time, that CSS has a strong synergistic effect with sorafenib in the inhibition of HCC cell proliferation.

To further explore the core compounds, potential target genes, and pathways of CSS in suppressing HCC, we established a drug‐compound‐target network and found that the top 10 active components were β‐sitosterol and flavonoids such as quercetin, kaempferol, luteolin and licochalcone A. Additionally, combined PPI network construction and transcriptome data analysis revealed three hub genes, viz. AKT1, MAPK3 and CASP3, which are not only potential targets of CSS against HCC, but also have prognostic potential in HCC. Numerous studies have reported the crucial role of AKT in cell survival owing to its significance in the PI3K/AKT/mTOR signalling pathway. As an isoform of AKT, AKT1 is widely expressed in the liver, and the accumulation of activated AKT1 is associated with poor survival in HCC.^18^, ^19^ Xu et al. reported that the loss of AKT1 completely prevents c‐Myc HCC formation in mice, and silencing of AKT1 in c‐Myc HCC cell lines strongly suppresses cell growth.^19^ Reduction of AKT1 expression using RNAi or natural compounds induces apoptosis and G1 cell cycle arrest in HCC.^20^, ^21^ In addition, studies have shown that drug resistance‐related genes like Y‐box binding protein 1 (YB‐1) and forkhead box M1 (FoxM1) promote sorafenib resistance in HCC cells by inducing AKT1 activity.^22^, ^23^ These studies suggest that targeting AKT1 may be the major mechanism by which CSS inhibits HCC progression and increases the efficacy of sorafenib therapy. Of note, the results of our molecular docking and SPR assays indicated potential binding between AKT1 and luteolin, which echoed a recent study that reported that luteolin could induce apoptosis by inhibiting the AKT pathway in HCC cells.^24^

Luteolin is a representative derivative of dihydroflavonoids found in many plants, including XF. Its structure consists of two aromatic rings connected by three carbon atoms in the center, which determine its multi‐biological activities and multi‐target mechanisms.^25^, ^26^ Here, we found that luteolin also acts on MAPK3 protein targets in addition to AKT1. Mitogen‐activated protein kinase 3 (MAPK3), also named ERK1, together with ERK2, mainly operates in mitogen‐activated signal transduction pathways and plays a prominent role in regulating malignant cell proliferation, migration, and invasion.^27^ ERK1/2 requires dual phosphorylation to activate and transmit signals from surface receptors to the nucleus to modulate oncogenic activity.^28^ Gao et al.^29^ reported that p‐ERK1 expression is closely related to the clinicopathological characteristics of patients with HCC, such as age, sex, pathological stage, tumour diameter, Child–Pugh classification, and serum levels of AFP, ALT, AST, PLT and ALB. There is a strong correlation between ERK1 expression and sorafenib resistance in liver cancer.^30^, ^31^ Therefore, we believe that AKT1, MAPK3, and their corresponding binding active components in CSS (e.g., luteolin) play critical roles in HCC, and it is necessary to study the interaction between them as well as their potential link with HCC.

Caspase‐3 is an executioner of caspases that plays an important role in apoptosis. It is indispensable for apoptotic chromatin condensation and DNA fragmentation in all cell types examined, as well as for the dismantling of the cell and the development of apoptotic bodies, and has thus become a primary target for anti‐cancer therapy.^32^, ^33^ Several plant isolates have been shown to stimulate caspase‐3‐mediated apoptosis‐induced cytotoxicity. Similarly, podophyllotoxin isolated from *Dysosma versipellis* induces cell death in lung cancer cells by downregulating the expression of caspase‐3, whereas, majoranolide isolated from *Mezilaurus crassiramea* induces caspase‐3‐mediated apoptosis‐induced cytotoxicity in human promyelocytic leukaemia cells.^33^ Our data indicate that licochalcone A may target caspase‐3 to suppress cell viability in HCC cells; however, this mechanism has not been reported in HCC to date, and further experiments are needed for verification.

Since KEGG pathway analysis implied the involvement of CSS targets in the immunological signalling pathway, we estimated the proportion of tumour‐infiltrating immune cells in low‐ and high‐risk patients with HCC using the CSS target three‐gene signature model. We found a significant enrichment of Treg cells and M0 macrophages in high‐risk patients. As Tregs and M0 macrophages are widely regarded as immunosuppressive cells,^34^, ^35^ our findings indicate a predominance of cancer‐promoting immune cells in the high‐risk group. In addition, the elevated expression of immune checkpoint proteins that facilitate immune escape has been observed in high‐risk patients with HCC. Thus, the poor prognosis of the high‐risk group may be related to the higher immunosuppression and lower immunoreactivity of the tumour microenvironment. This finding also suggests that the CSS regimen investigated in this study may be more effective in patients with HCC with a higher risk score. Therefore, the risk scores derived from the CSS target three‐gene signature model may be useful in guiding clinical immunotherapy.

Overall, our study analysed the synergistic effects of CSS and sorafenib in reducing HCC cell growth, and identified the core compounds and hub targets of CSS for the treatment of HCC. Compared with previous studies, we developed a LASSO‐Cox prognostic regression model based on three hub targets and analysed the immune effects of CSS. We speculate that CSS may regulate the activity of immune‐related factors in the tumour microenvironment, reverse immune escape, enhance immune responses through AKT1, MAPK3, and CASP3, and synergistically alleviate HCC. Our findings help to elucidate the complex mechanism of CSS against HCC and suggest a potential clinical application of CSS in HCC adjuvant therapy. However, our study has some limitations. First, the acquisition of bioactive ingredients contained in CSS was based on existing databases rather than liquid chromatography, mass spectrometry, or other analytical methods. Second, the information in the databases is inclined towards hot topics and a limited number of small‐molecule compounds and drug targets. Third, although the effect of CSS combined with sorafenib was preliminarily identified, this study lacked mechanistic verification. Therefore, follow‐up pharmacological experiments and related molecular biology experiments are required to further clarify the bioactive ingredients of CSS and verify the regulatory synergistic mechanism of CSS and sorafenib on key targets in HCC. Further clinical validation studies are required to confirm the prognostic efficiency of the developed CSS target three‐gene signature model.

## AUTHOR CONTRIBUTIONS

**Jia‐heng Xing:** Data curation (equal); formal analysis (equal); resources (equal); visualization (equal); writing – original draft (equal); writing – review and editing (equal). **Ru‐xue Tan:** Data curation (equal); funding acquisition (equal). **Fei‐er Huang:** Investigation (equal); resources (equal); visualization (equal); writing – review and editing (equal). **Nan Tian:** Conceptualization (equal); funding acquisition (equal); methodology (equal); project administration (equal); supervision (equal); writing – review and editing (equal).

## CONFLICT OF INTEREST STATEMENT

The authors declare that they have no known competing financial interests or personal relationships that could have appeared to influence the work reported in this paper.

## Data Availability

Publicly available datasets were analysed in this study. The data is available at TCGA, TCMSP, GeneCard and OMIM database. All analysed or generated data are included in the article.
